# FLI1 in PBMCs contributes to elevated inflammation in combat-related posttraumatic stress disorder

**DOI:** 10.3389/fpsyt.2024.1436690

**Published:** 2024-07-30

**Authors:** Pengfei Li, Liu Liu, Shufeng Liu, Zhongyang Lu, Perry V. Halushka, Sara J. Sidles, Amanda C. LaRue, Zhewu Wang, Hongkuan Fan

**Affiliations:** ^1^ Department of Pathology and Laboratory Medicine, Medical University of South Carolina, Charleston, SC, United States; ^2^ Research Service, Ralph H. Johnson Department of Veterans Affairs Health Care System, Charleston, SC, United States; ^3^ Department of Psychiatry, Medical University of South Carolina, Charleston, SC, United States; ^4^ Department of Medicine, Medical University of South Carolina, Charleston, SC, United States; ^5^ Department of Pharmacology, Medical University of South Carolina, Charleston, SC, United States

**Keywords:** FLI1, inflammation, PTSD, PBMC, antisense oligonucleotides

## Abstract

Post-traumatic stress disorder (PTSD) is a debilitating psychiatric condition with significant public health implications that arise following exposure to traumatic events. Recent studies highlight the involvement of immune dysregulation in PTSD, characterized by elevated inflammatory markers. However, the precise mechanisms underlying this immune imbalance remain unclear. Previous research has implicated friend leukemia virus integration 1 (FLI1), an erythroblast transformation-specific (ETS) transcription factor, in inflammatory responses in sepsis and Alzheimer’s disease. Elevated FLI1 levels in peripheral blood mononuclear cells (PBMCs) have been linked to lupus severity. Yet, FLI1’s role in PTSD-related inflammation remains unexplored. In our study, PBMCs were collected from Veterans with and without PTSD. We found significantly increased FLI1 expression in PBMCs from PTSD-afflicted Veterans, particularly in CD4^+^ T cells, with no notable changes in CD8^+^ T cells. Stimulation with LPS led to heightened FLI1 expression and elevated levels of inflammatory cytokines IL-6 and IFNγ in PTSD PBMCs compared to controls. Knockdown of FLI1 using Gapmers in PTSD PBMCs resulted in a marked reduction in inflammatory cytokine levels, restoring them to control group levels. Additionally, co-culturing PBMCs from both control and PTSD Veterans with the human brain microglia cell line HMC3 revealed increased inflammatory mediator levels in HMC3. Remarkably, HMC3 cells co-cultured with PTSD PBMCs treated with FLI1 Gapmers exhibited significantly lower inflammatory mediator levels compared to control Gapmer-treated PTSD PBMCs. These findings suggest that suppressing FLI1 may rebalance immune activity in PBMCs and mitigate microglial activation in the brain. Such insights could provide novel therapeutic strategies for PTSD.

## 1 Introduction

Posttraumatic stress disorder (PTSD) is a trauma and stressor-related disorder that emerges following traumatic events such as combat (1, 2). Globally, this mental health condition affects approximately 5.6% of those exposed to trauma (3). Among individuals exposed to military combat, the prevalence can soar to as high as 11.1%-30% (4). Recent studies highlight that PTSD is associated with systemic inflammation and neuroinflammation (1, 5). Combat-exposed subjects with PTSD had significantly higher circulating inflammatory mediators, including IL-6 and IFNγ, compared to combat-exposed subjects without PTSD (6, 7). Mechanistically, the hyper-reactivity of gene alteration in peripheral blood mononuclear cells (PBMCs) may contribute significantly to the elevated systemic inflammation in combat veterans with PTSD (8, 9). Circulating inflammatory mediators can traverse the blood-brain barrier, potentially triggering neuroinflammation (10–12). Furthermore, animal models of PTSD have demonstrated elevated levels of inflammatory mediators such as IL-6 and IFNγ in the brain, which correlate with the development and persistence of PTSD (1, 13). Hence, a deeper understanding of the associated inflammation in PTSD may provide implications for PTSD prevention and treatment.

Friend leukemia virus integration 1 (FLI1), an ETS transcription factor, regulates a broad spectrum of biological processes, including cancer development (14, 15), fibrosis, and inflammation (16–21). FLI1 is expressed in endothelial cells, macrophages, B cells, and T cells, where it regulates the expression of several critical inflammatory mediators, including IL-6 and IFNγ (19, 20, 22). Moreover, FLI1 plays a pivotal role in immune cell development, proliferation, activation, and migration, including T cells, B cells, and monocytes/macrophages (22, 23). FLI1 can also affect the function of immune cells by regulating cytokines and chemokines and contributes to inflammatory responses in various inflammatory diseases, including sepsis, Alzheimer’s disease, systemic sclerosis, lupus, and graft-versus-host disease (22–25). Moreover, Georgiou et al. demonstrated a potential correlation between the overexpression of FLI1 in PBMCs and the severity of lupus in patients (26). Furthermore, our previous findings indicated that FLI1 contributes to the neuroinflammation in Alzheimer’s disease (24). However, the specific role of FLI1 and its impact on PBMCs and associated inflammation in PTSD remain elusive.

This constellation of findings led us to explore the role of FLI1 in PBMCs and associated inflammation in PTSD. To our knowledge, there has been no investigation of potential links between FLI1 and PTSD. Hence, the present study sought to examine the role of FLI1 in PTSD within a group of combat veterans, and we hypothesized that increased FLI1 in PBMCs contributes to systemic inflammation and neuroinflammation in PTSD.

## 2 Methods

### 2.1 Human donors

Twelve combat veterans in the control group and twelve combat veterans in a group diagnosed with PTSD, recruited through the Ralph H. Johnson Veterans Affairs Medical Center (VAMC), were enrolled in this study. The Medical University of South Carolina Institutional Review Boards approved the research protocol. The study’s brief description and voluntary nature were explained to participants by a trained research assistant, and interested participants were screened to determine eligibility. Written informed consent was obtained from all participants before the formal interview.

### 2.2 Inclusion/exclusion criteria for participants

Inclusion criteria included a history of combat exposure, as evidenced by an official service record and report of combat exposure during the interview. In order to match the trauma-exposure severity between the PTSD subjects and controls, the majority of participants in the second half of this study were required to have a Combat Exposure Scale (CES) score of 8 or above. The CES is a 4-point likert scale that measures exposure to combat experiences and can range from 0–41 points. The total score is calculated by adding weighted scores from questions about combat exposure, such as being under enemy fire, seeing someone hit by rounds, or firing at the enemy. Score 8 or above is considered as beyond mild trauma exposure which may satisfy PTSD diagnosis. Exclusion criteria included: current or lifetime DSM-IV schizophrenia, other psychotic disorders, bipolar disorder, and active substance abuse or dependence in the past six months. Individuals with a history of substance abuse and dependence were included if the last use of the substance was over six months before enrollment. Control subjects were selected to be free of Axis I disorders. Additional information about control and PTSD study participants has been previously described (27–29). Patient demographics are listed in **Table 1**.

**Table 1:** Demographic characteristics of cohort.

N=24.	Combat Veterans with PTSD	Combat Veterans without PTSD
Subject size	12	12
Sex, Females (%)	5 (41.6%)	2 (16.7%)^*^
Age ± SEM (years)	37.2 ± 2.3	44.5 ± 2.7^#^
Race (%)		
White	5 (41.6%)	7 (58.3%)
Black	7 (58.3%)	3 (25%)
Other	N/A	2 (16.7%)
CES	23.3 ± 2.2	22.9 ± 2.1^$^
Severity Scale	35.6 ± 2.0	12.8 ± 2.0^&^

SEM, standard error of the mean; N/A, not applicable; CES, Combat Exposure Scale. *P=0.3707, Chi-square test was used; ^#^P=0.0504, Student’s t-test was used; ^$^P=0.9079, Student’s t-test was used; ^&^P<0.0001, Student’s t-test was used.

### 2.3 Isolation, activation, culture, and treatment of PBMCs

PBMCs were purified from fresh whole blood of Control and PTSD subjects using ACCUSPIN System-Histopaque-1077 (Sigma) as described (30). The isolated PBMCs were then activated with Dynabeads™ Human T-Expander CD3/CD28 (ThermoFisher Scientific) at a bead-to-cell ratio of 1:1 and maintained in RPMI-1640 medium (Gibco, USA) supplemented with 10% fetal bovine serum (FBS; Gibco), 1% penicillin/streptomycin (Gibco, USA), and 100 U/ml human IL-2 (Peprotech) in a 5% CO_2_ incubator at 37°C for five days. FLI1 mRNA and protein levels in these activated cells (1x10^7^) were assessed using Real-Time PCR and Western Blot, respectively.

In another set of experiments, CD3/CD28-activated PBMCs were treated with either Control or FLI1 Gapmers (Qiagen) for 24 hours, followed by incubation with LPS (200 ng/ml, Sigma) for 24 hours. Total RNA and supernatant were collected from 4x10^6^ PBMCs for further analysis. Moreover, these treated PBMCs were centrifuged at 300 g for 5 minutes and utilized in co-culture experiments with HMC3.

### 2.4 Isolation, culture, and treatment of CD4^+^ and CD8^+^ T cell

According to the manufacturer’s instructions, CD4^+^ and CD8^+^ T cells were isolated from CD3/CD28-activated PBMCs using CD4 and CD8 magnetic beads (Miltenyi Biotec Inc.). FLI1 mRNA and protein levels within these T cells were detected using Real-Time PCR and Western Blot.

### 2.5 HMC3 culture

HMC3 was obtained from the American Type Culture Collection (ATCC) and cultured in EMEM (ATCC) supplemented with 10% FBS (Gibco) and 1% penicillin/streptomycin (Gibco) in a CO_2_ incubator with 5% CO_2_.

### 2.6 Establishment of co-culture model

12-well plates and 12-mm diameter transwell inserts with 0.4-μm pore size (Corning) were employed for co-culture experiments. Initially, HMC3 cells (1×10^6^ cells/well) in 1 mL complete medium were seeded into the 12-well plates and allowed to adhere for 6 hours. Subsequently, treated PBMCs (4×10^6^ cells/well) in 500 μL complete media supplemented with 100 U/ml human IL-2 (Peprotech) were added into the upper chamber of the transwell inserts for each of the 12 wells containing HMC3. The ratio of HMC3 to PBMCs was 1:4. These cells were co-cultured in a 5% CO_2_ incubator at 37 °C for 24 h. Supernatants from the HMC3 cells were collected for ELISA analysis.

### 2.7 ELISA

The levels of IL-6 and IFNγ in the cell supernatant were measured using an IL-6 or IFNγ Human ELISA Kit (ThermoFisher Scientific), following the manufacturer’s instructions.

### 2.8 Real-Time RT-PCR

Total RNA was extracted from PBMCs, CD4^+,^ or CD8^+^ T cells using the RNeasy Plus Mini Kit (Qiagen). cDNA was synthesized with the High-Capacity cDNA Reverse Transcription Kit (Applied Biosystems). Quantitative real-time PCR was performed using the SYBR Green PCR Kit (Qiagen) and CFX96 Real-Time PCR system (Bio-Rad). Primers used in this study were purchased from Qiagen. Data were analyzed with 2^−ΔΔCt^ value calculation using GAPDH for normalization.

### 2.9 Western Blot

PBMCs, CD4^+,^ or CD8^+^ T cells were lysed with ice-cold RIPA lysis buffer (Abcam) containing protease and phosphatase inhibitors (Cell Signaling). All lysed samples were kept on ice for 30 minutes and centrifuged for 10 minutes at 4 °C at 10,000×*g*. The cell lysates were collected, and protein concentrations were measured using a DC protein assay (Bio-Rad). A total of 20 μg of protein was loaded into each lane for western blot. Primary antibodies, including the anti-FLI1 (Abcam) and the anti-GAPDH antibodies (Cell Signaling), were used at 1:1000 dilution, and peroxidase-labeled secondary antibody (Thermofisher Scientific) was used at 1: 10000 dilutions. The immunoreactive protein bands were visualized using an ECL detection kit (GE Healthcare) and analyzed using the NIH ImageJ software.

### 2.10 Data analysis

Statistical significance was determined by analysis of variance (ANOVA) or Student’s t-test using GraphPad Prism software. For ANOVA, “Tukey’s Multiple Comparison Test” was used for *post-hoc* comparisons. A value of p < 0.05 was considered statistically significant.

## 3 Results

### 3.1 Increased FLI1 in the plasma PBMCs and CD4^+^ T cells in patients with PTSD

**Figure 1:**
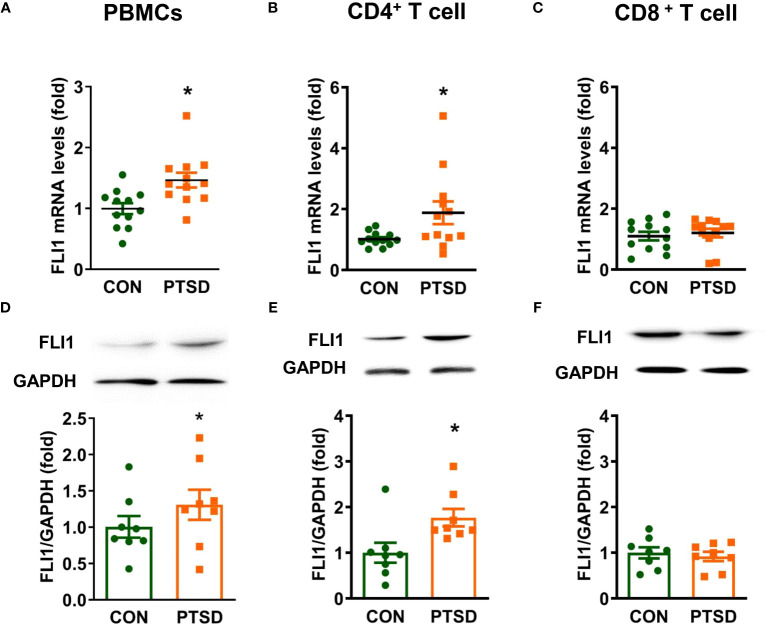
Elevated FLI1 levels in PBMCs and CD4^+^ T cells in combat-related PTSD subjects. PBMCs were purified from fresh whole blood of combat veterans with or without PTSD and activated with CD3/CD28 dynabeads. CD4^+^ and CD8^+^ T cells were separated from CD3/CD28-activated PBMCs using CD4 and CD8 magnetic beads, respectively. **(A-C)** FLI1 mRNA levels in PBMCs, CD4^+^, and CD8^+^ T cells were detected by Real-time PCR. N=12. ^*^p < 0.05 compared to the control group. **(D-F)** Protein levels of FLI1 in PBMCs, CD4^+^, and CD8^+^ T cells were determined by Western Blot. N=8. ^*^p < 0.05 compared to the control group.

To investigate the potential involvement of FLI1 in PTSD, we initially determined FLI1 expression levels in individuals exposed to combat with PTSD as compared to individuals exposed to combat without PTSD. PBMCs were isolated from fresh whole blood samples obtained from combat-exposed Control and PTSD subjects, followed by activation with Dynabeads™ Human T-Expander CD3/CD28. Our findings indicate a significant increase in FLI1 mRNA levels, as determined by real-time PCR, in the plasma PBMCs of PTSD patients (n = 12 per group, **Figure 1A**; p < 0.05) compared to control patients. As T cells constitute a major subset of activated PBMCs, we subsequently isolated CD4^+^ and CD8^+^ T cells from these PBMCs. Elevated FLI1 mRNA levels were observed in CD4^+^ T cells from PTSD patients (n = 12 per group, **Figure 1B**; p < 0.05), whereas no significant difference was detected in CD8^+^ T cells (n = 12 per group, **Figure 1C**; p > 0.05). Similarly, Western blot analysis revealed a significant increase in FLI1 protein levels in PBMCs and CD4^+^ T cells from PTSD patients (n = 8 per group, **Figures 1D–E**; p < 0.05). However, no difference in FLI1 protein levels was observed in CD8^+^ T cells between the two groups (n = 8 per group, **Figure 1F**; p > 0.05). Demographic information for human subjects involved in the study is provided in **Table 1**.

### 3.2 FLI1 contributes to inflammatory response in LPS-stimulated PBMCs

**Figure 2:**
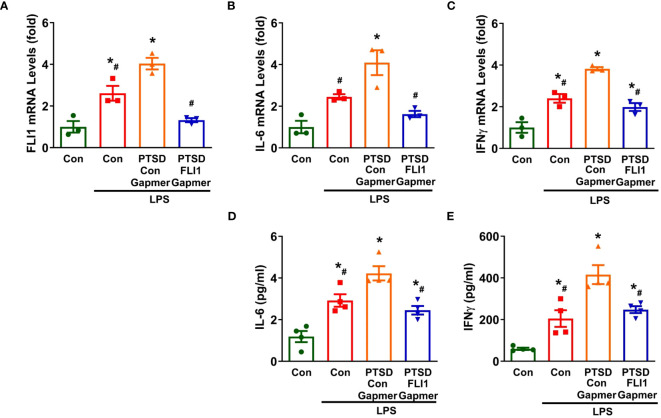
Inhibition of FLI1 reduces LPS-stimulated IL-6 and IFNγ production by PBMCs in combat-related PTSD subjects. PBMCs activated with CD3/CD28 from combat veterans, with or without PTSD, were treated with control or FLI1 Gapmer for 24 hours, followed by additional stimulation with LPS (200 ng/ml) for another 24 hours. The mRNA levels of FLI1 **(A)**, IL-6 **(B)**, and IFNγ **(C)** in PBMCs were assessed. Data were obtained from three control or PTSD subjects, with three independent experiments. *p < 0.05 compared to the non-stimulated control PBMC group, while ^#^p < 0.05 compared to the PTSD PBMCs + control Gapmer group. Statistical significance was determined by ANOVA (**A**, F=26.69, P=0.0002, R^2^ = 0.9092; **B**, F=14.92, P=0.0012, R^2^ = 0.8484; **C**, F=36.69, P<0.0001, R^2^ = 0.9322). Additionally, the production of IL-6 **(D)** and IFNγ **(E)** in the supernatant was measured using ELISA. Data were collected from four control or PTSD subjects, with three independent experiments. *p < 0.05 compared to the non-stimulated control PBMC group, while ^#^p < 0.05 compared to the PTSD PBMCs + control Gapmer group. Statistical significance was determined by ANOVA (**D**, F=19.6, P<0.0001, R^2^ = 0.8305; **E**, F=21.54, P<0.0001, R^2 =^ 0.8434).

Emerging evidence from clinical and basic studies suggests that PTSD is associated with systemic and central nervous system (CNS) inflammation (5, 31). To further explore the role of FLI1 in inflammation mediated by PBMCs, activated PBMCs by Dynabeads™ Human T-Expander CD3/CD28 were treated with Control or FLI1 Gapmer for 24 hours, followed by stimulation with LPS for an additional 24 hours. Post-LPS exposure, PBMCs derived from PTSD patients displayed a notable increase in FLI1 expression compared to those from control subjects; however, this increase was significantly suppressed with the knockdown of FLI1 via FLI1 Gapmer (n=3 per group from three independent experiments, **Figure 2A**; p < 0.05). Correspondingly, heightened mRNA levels of IL-6 and IFNγ were observed in PBMCs from PTSD patients in contrast to control subjects (n=3 per group from three independent experiments, **Figures 2B, C**; p < 0.05). These elevated levels were markedly attenuated in the FLI1 Gapmer-treated group (n=3 per group from three independent experiments, **Figures 2B, C**; p < 0.05). Analysis of IL-6 and IFNγ protein levels in the cell supernatant showed that PBMCs from PTSD patients exhibited increased levels of these inflammatory mediators, which were significantly reduced upon FLI1 Gapmer treatment (n=4 per group from three independent experiments, **Figures 2D, E**; p < 0.05).

### 3.3 FLI1 contributes to inflammatory response in microglia via PBMCs in the co-culture model

**Figure 3:**
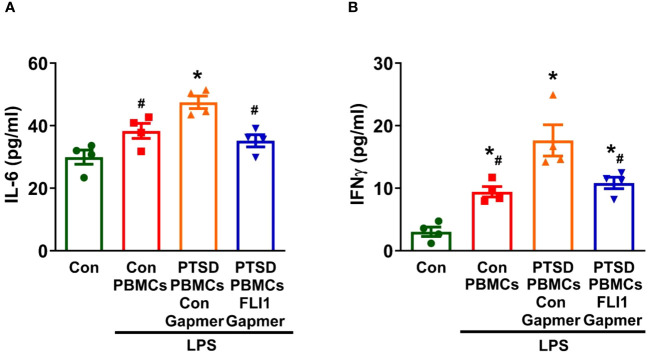
Inhibition of PBMC FLI1 inhibits inflammatory mediators produced by human brain microglia after co-culture with LPS-stimulated PBMCs. PBMCs activated with CD3/CD28 from combat veterans, with or without PTSD, were treated with control or FLI1 Gapmer for 24 hours, followed by additional stimulation with LPS (200 ng/ml) for another 24 hours. HMC3 cells were cultured alone or co-cultured with these treated PBMCs at a 1:4 ratio in a membrane insert for 24 hours. The production of IL-6 **(A)** and IFNγ **(B)** in the supernatant from HMC3 cells was assessed using ELISA. Data were collected from four control or PTSD subjects, with three independent experiments. *p < 0.05 compared to the control HMC3 group, while ^#^p < 0.05 compared to the HMC3 group co-cultured with control Gapmer-treated PTSD PBMCs. Statistical significance was determined by ANOVA (**A**, F=11.63, P=0.0007, R^2^ = 0.7441; **B**, F=17.11, P=0.0001, R^2^ = 0.8105).

Microglial activation has been observed in PTSD, correlating with the accumulation of inflammatory mediators in the brain (1, 2). To further explore the role of PBMC FLI1 in CNS inflammation, we conducted co-cultures of human microglia (HMC3) with PBMCs for 24 hours at a ratio of 4 PBMCs per microglia. Co-culture with LPS-stimulated PBMCs from Control subjects slightly increased IL-6 production in the supernatant of HMC3 (n=4 per group from three independent experiments, **Figure 3A**; p > 0.05) compared to the HMC3 alone group. Interestingly, IL-6 secretion was significantly elevated in HMC3 cells co-cultured with LPS-stimulated PBMCs from PTSD patients; this increase was inhibited by knockdown of FLI1 via FLI1 Gapmer (n=4 per group from three independent experiments, **Figure 3A**; p < 0.05). Similarly, co-culture with LPS-stimulated PBMCs from control subjects led to a moderate increased in IFNγ production by HMC3 cells, whereas co-culture with LPS-stimulated PBMCs from PTSD subjects led to a significant increase in IFNγ production (n=4 per group from three independent experiments, **Figure 3B**; p < 0.05). The elevated levels of IFNγ were significantly attenuated in the FLI1 Gapmer-treated group (n=4 per group from three independent experiments, **Figure 3B**; p < 0.05).

## 4 Discussion

The role of FLI1 in inflammation associated with PTSD remains unexplored to date. Our current study demonstrated several significant discoveries. Firstly, we observed elevated levels of FLI1 in plasma PBMCs, particularly within CD4^+^ T cells, in individuals who have combat-related PTSD. Secondly, our study indicates that FLI1 contributes to a heightened inflammatory response in LPS-activated PBMCs from PTSD patients compared to control patients. Lastly, soluble factors derived from PTSD PBMCs trigger activation of brain microglia, and expression of FLI1 in PBMCs is critical for this process. These findings shed light on the importance of FLI1 function within PBMCs and underscore its pivotal role in modulating both systemic and CNS inflammation associated with PTSD. Consequently, FLI1 emerges as a promising biomarker or therapeutic target for PTSD.

Our study is the first to demonstrate that FLI1 levels are elevated in plasma PBMCs and may contribute to systemic inflammation in combat-related PTSD. PTSD, a trauma-related mental disorder, has increasingly been associated with heightened systemic inflammation, which may play a role in the link between trauma and the development of PTSD (10, 32, 33). Combat-exposed individuals with PTSD exhibit higher circulating levels of inflammatory mediators such as IL-6 and IFNγ compared to those exposed to combat but without PTSD (6, 34, 35). PBMCs have been shown to play a significant role in elevating systemic inflammatory mediators in combat veterans with PTSD (8, 9). The spontaneous production of inflammatory mediators, including IL-6, by isolated PBMCs is significantly higher in PTSD patients compared to control patients and correlates with PTSD symptom severity (36). Previously, elevated FLI1 levels in PBMCs were observed in lupus patients and were related to disease severity (26). In our study, we show for the first time that FLI1 is increased in plasma PBMCs from combat veterans with PTSD compared to those without PTSD at the mRNA and protein level (**Figure 1**). Interestingly, patients previously diagnosed with PTSD showed twice the likelihood of subsequent systemic lupus erythematosus (SLE) diagnosis (37). Therefore, elevated FLI1 levels in PBMCs among PTSD patients may partially contribute to lupus development. We found that in LPS-stimulated PBMC, mRNA expression and secreted levels of IL-6 and IFNγ were significantly increased in combat veterans with PTSD compared to combat controls (**Figure 2**). Importantly, inhibition of FLI1 in PTSD PBMCs via FLI1 Gapmer restored IL-6 and IFNγ levels to those from combat controls (**Figure 2**). Collectively, these findings provide the first evidence that elevated FLI1 in circulating PBMCs may contribute to systemic inflammation associated with PTSD.

Beyond systemic inflammation, we also show that FLI1 may contribute to CNS inflammation via PBMCs in combat-related PTSD. In animal models of PTSD, elevated levels of pro-inflammatory cytokines have been detected in key brain regions, including the hippocampus, amygdala, and prefrontal cortex (31, 38, 39). This phenomenon suggests a link between peripheral inflammation, mediated by PBMCs, and neuroinflammation, facilitated by the activation of microglia and astrocytes through cytokine transport across the blood-brain barrier (5, 10–12, 36, 40). Further supporting this notion, Tina et al. demonstrated that co-culture with activated PBMCs led to astrocyte upregulation of pro-inflammatory genes and proteins (41). In this study, we show that brain microglia co-cultured with activated PBMCs from PTSD patients secrete increased levels of inflammatory mediators. Intriguingly, inhibition of FLI1 expression in PBMCs via FLI1 Gapmer resulted in a marked reduction of this pro-inflammatory response in microglia (**Figure 3**). Previously activated microglia have been shown to promote neuroinflammation and modulate the stress response in the brain in animal models of PTSD (5, 42–44). Our findings suggest that the observed increase in FLI1 expression in PTSD PBMCs (**Figure 1**) may contribute to elevated systemic inflammatory mediators and exacerbate inflammation within the brain by activating resident immune cells, such as microglia. This intricate relationship underscores the multifaceted role of FLI1 in orchestrating immune responses across systemic and CNS compartments, offering novel insights into the pathogenesis of PTSD-associated neuroinflammation and potential therapeutic avenues for intervention.

Importantly, our study shows that FLI1 expression is selectively heightened in CD4^+^ T cells from combat veterans with PTSD (**Figure 1**). Previous research has shown an increase in pro-inflammatory CD4^+^ T-cell subsets, including Th1 and Th17 cells, alongside a decreased proportion of anti-inflammatory Tregs in PTSD cases (45–47). Schutt et al. elucidated FLI1’s pivotal role in alloreactive CD4^+^ T cell activation and differentiation in Graft-versus-host disease (22), while its involvement in Treg induction in systemic sclerosis has been highlighted in other studies (48). Our findings support these previous studies and reveal increased FLI1 levels in CD4^+^ T cells from patients with combat-related PTSD (**Figure 1**). Future studies will focus on the role of FLI1 in CD4^+^ T cell activation and differentiation in the context of PTSD.

This study has several limitations that warrant consideration. Firstly, our determination of FLI1 levels in PBMCs and CD4^+^ T cells was conducted within a small cohort of PTSD patients. However, to enhance the robustness of our findings, further validation studies involving larger cohorts of individuals with PTSD are imperative. Secondly, there is a need for additional investigation into FLI1 levels within specific subsets of T cells and PBMCs in PTSD patients. These future studies will help to elucidate the precise role of FLI1 in modulating CD4^+^ T cell function and its potential correlation with the severity of inflammation in PTSD.

In summary, our study has unveiled a novel association between FLI1 expression in PBMCs and inflammation linked to PTSD. These findings hold promise for the identification of novel biomarkers for PTSD and may pave the way for innovative therapeutic strategies.

## Data availability statement

The original contributions presented in the study are included in the article/supplementary material. Further inquiries can be directed to the corresponding author.

## Ethics statement

The studies were conducted in accordance with the local legislation and institutional requirements of The Medical University of South Carolina Institutional Review Boards. The human samples used in this study were acquired from our previous study (IRB #: Pro00063496. Title: Dysregulated Immune State in PTSD Contributes to Microglial Inflammation) for which ethical approval was obtained. Written informed consent for participation was not required from the participants or the participants’ legal guardians/next of kin in accordance with the national legislation and institutional requirements.

## Author contributions

PL: Conceptualization, Data curation, Formal analysis, Investigation, Methodology, Resources, Software, Validation, Visualization, Writing – original draft, Writing – review & editing. LL: Data curation, Writing – review & editing. SL: Data curation, Writing – review & editing. ZL: Data curation, Writing – review & editing. PH: Writing – review & editing. SS: Writing – review & editing, Methodology. AL: Methodology, Writing – review & editing. ZW: Writing – review & editing, Resources. HF: Conceptualization, Data curation, Funding acquisition, Investigation, Methodology, Project administration, Resources, Supervision, Writing – review & editing.
